# Evaluation of the antibacterial power and biocompatibility of zinc oxide nanorods decorated graphene nanoplatelets: new perspectives for antibiodeteriorative approaches

**DOI:** 10.1186/s12951-017-0291-4

**Published:** 2017-08-01

**Authors:** Elena Zanni, Erika Bruni, Chandrakanth Reddy Chandraiahgari, Giovanni De Bellis, Maria Grazia Santangelo, Maurizio Leone, Agnese Bregnocchi, Patrizia Mancini, Maria Sabrina Sarto, Daniela Uccelletti

**Affiliations:** 1 Department of Biology and Biotechnology C. Darwin, Sapienza University of Rome, Piazzale Aldo Moro 5, Rome, Italy; 2 Research Center on Nanotechnology Applied to Engineering of Sapienza (CNIS), SNNLab, Sapienza University of Rome, Piazzale Aldo Moro 5, Rome, Italy; 3 Department of Astronautical, Electrical and Energy Engineering, Sapienza University of Rome, Via Eudossiana 18, Rome, Italy; 4 Department of Physics and Chemistry, University of Palermo, Palermo, Italy; 5 Department of Experimental Medicine, Sapienza University of Rome, Viale Regina Elena 324, Rome, Italy

## Abstract

**Background:**

Nanotechnologies are currently revolutionizing the world around us, improving the quality of our lives thanks to a multitude of applications in several areas including the environmental preservation, with the biodeterioration *phenomenon* representing one of the major concerns.

**Results:**

In this study, an innovative nanomaterial consisting of graphene nanoplatelets decorated by zinc oxide nanorods (ZNGs) was tested for the ability to inhibit two different pathogens belonging to bacterial *genera* frequently associated with nosocomial infections as well as biodeterioration phenomenon: the Gram-positive *Staphylococcus aureus* and the Gram-negative *Pseudomonas aeruginosa*. A time- and dose-dependent bactericidal effect in cell viability was highlighted against both bacteria, demonstrating a strong antimicrobial potential of ZNGs. Furthermore, the analysis of bacterial surfaces through Field emission scanning electron microscopy (FESEM) revealed ZNGs mechanical interaction at cell wall level. ZNGs induced in those bacteria deep physical damages not compatible with life as a result of nanoneedle-like action of this nanomaterial together with its nanoblade effect. Cell injuries were confirmed by Fourier transform infrared spectroscopy, revealing that ZNGs antimicrobial effect was related to protein and phospholipid changes as well as a decrease in extracellular polymeric substances; this was also supported by a reduction in biofilm formation of both bacteria. The antibacterial properties of ZNGs applied on building-related materials make them a promising tool for the conservation of indoor/outdoor surfaces. Finally, ZNGs nanotoxicity was assessed in vivo by exploiting the soil free living nematode *Caenorhabditis elegans*. Notably, no harmful effects of ZNGs on larval development, lifespan, fertility as well as neuromuscular functionality were highlighted in this excellent model for environmental nanotoxicology.

**Conclusions:**

Overall, ZNGs represent a promising candidate for developing biocompatible materials that can be exploitable in antimicrobial applications without releasing toxic compounds, harmful to the environment.

**Electronic supplementary material:**

The online version of this article (doi:10.1186/s12951-017-0291-4) contains supplementary material, which is available to authorized users.

## Background

Nowadays, an ever-growing interest is focused on nanoscience that works with and/or creates promising materials characterized by nanostructured dimensions. Nanotechnologies have extensively been developed in the last years, expanding more and more the range of possible applications. At the present, nanotechnology has implications in a plethora of areas including medicine, food industry and environmental field, depending on specific nanomaterial features such as mechanical, thermal and chemical properties as well as large surface area [1–3]. Nevertheless, the antimicrobial power together with optical/light properties make some nanostructures particularly helpful in applications involved in the conservation of cultural heritage and/or building construction. In fact, historic buildings need to be preserved avoiding the risk of biodeterioration. Such process lead to unpleasant alteration of the material determined by the metabolism of bacteria, fungi, algae and lichens [4, 5]. Biodeteriorative activities determine severe damages to architectural surfaces, church frescoes or wall paintings that are found in catacombs and caverns. Among the bacterial isolates derived from wall paintings, *Pseudomonas* and *Staphylococcus* genera are the most predominant together with *Bacillus*, *Streptomyces* and *Mycobacterium* [6]. The formation of bacterial biofilm on construction material plays a key role in the possible occurrence of pathogen infections in nosocomial environments as well as in building biodeterioration [7, 8]. In fact, bacterial growth on wall surface as well as on medical devices represents a severe concern in the health care system, taking into account that bacteria are becoming multiresistant to antibiotics. From this perspective, the development of surfaces able to kill or inhibit bacterial growth without the use of antibiotics/drugs is attracting a great interest, and new wall paint and coatings, containing nanoparticles that possess antimicrobial activity, represent an emerging approach in order to prevent both the spread of nosocomial infections and biodeteriorative activity [9].

Among nanomaterials, great interest is currently addressed to the synthesis and development of graphene-based nanocomposites as reported in [10–13]. In particular, decoration of graphene with metal oxide offers unique properties that extensively broaden its application in chemical, medical and pharmaceutical fields [14, 15].

Several studies reported impressive antimicrobial power for metal oxide-based nanoparticles [16, 17]. Moreover, it has been possible to grow ZnO nanostructures onto graphene, so that decoration or functionalization was typically achieved only over the exposed surface of graphene. In our recent study, the synthesis of ZnO nanorods (ZnO-NRs) with controlled shape and density onto unsupported multilayer graphene flakes (also known as graphene nanoplatelets GNPs) was reported [18].

These zinc oxide nanorods-decorated graphene nanoplatelets (ZNGs) were characterized by the ability to kill the bacterium causing dental caries, namely *Streptococcus mutans*. ZNGs were found to efficiently kill and to control *S. mutans* cells by inhibiting both planktonic and biofilm growth [19]. This hybrid nanomaterial combines the remarkable electrical and antimicrobial properties offered by GNPs together with optical features and the highly effective killer action against both Gram-positive and Gram-negative bacteria of ZnO-NRs. Moreover, the characteristic grey color of graphene based nanomaterials is mitigated by ZnO whitening effect, making this hybrid nanostructure a promising candidate for the development of novel nanofiller-based wall paint in the field of building construction and cultural heritage.

Herein, ZNGs were used to inhibit two pathogens belonging to genera frequently associated to biodeterioration: the Gram-positive *Staphylococcus aureus* and the Gram-negative *Pseudomonas aeruginosa*; a mechanical mode of action against both bacteria has been suggested. Environmental nanotoxicity was assessed through the soil free-living nematode *Caenorhabditis elegans*.

## Methods

### Production of nanostructures and suspensions

**Fig. 1:**
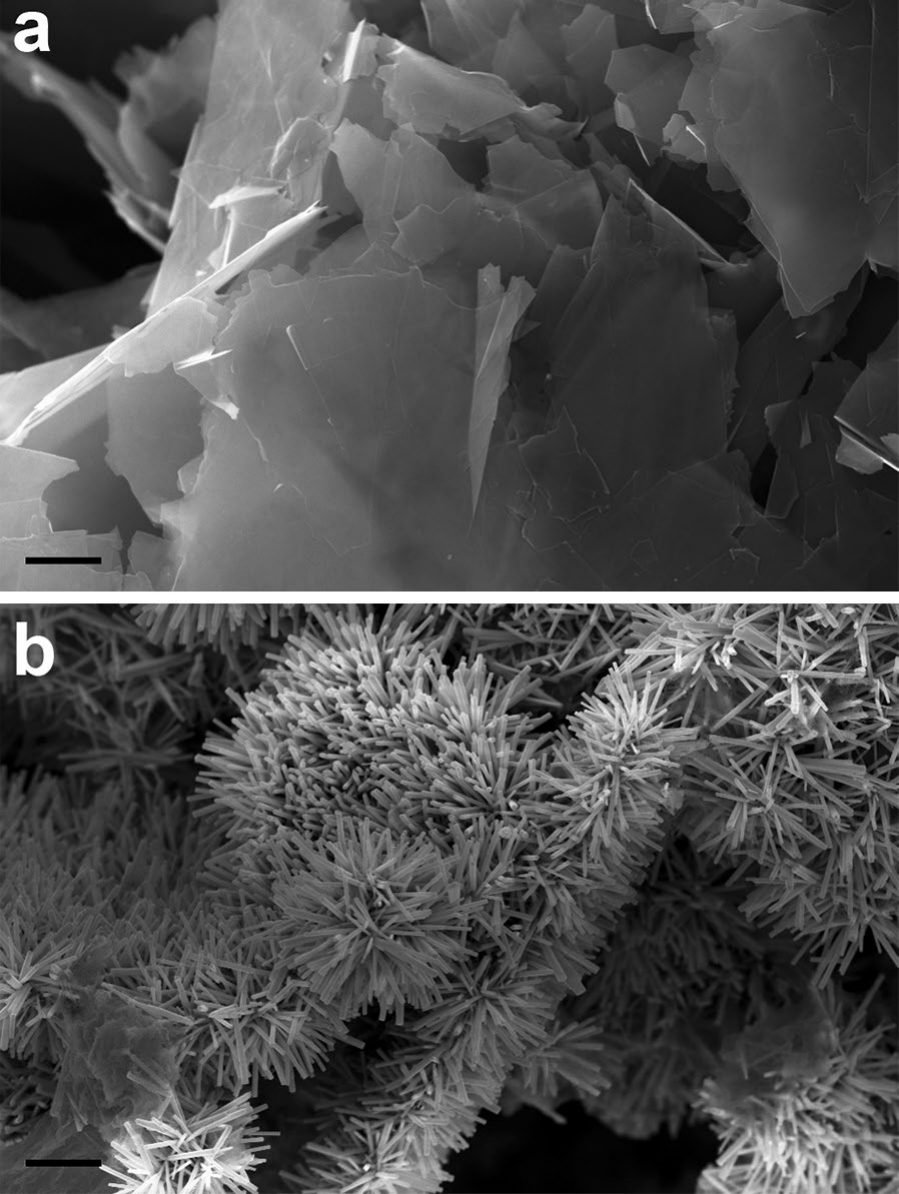
Field emission scanning electron microscopy (FE-SEM) images of **a** pristine graphene nanoplatelets (GNPs) and **b** ZnO-NRs-decorated GNPs (ZNG) (*scale bar* 1 µm)

Zinc oxide nanorods decorated graphene nanoplatelets were produced by a simple hydrothermal method as described in Chandraiahgari et al. [18]. Briefly, GNPs were derived through a solvothermal exfoliation process as described previously in Rago et al. [20]. Thereafter, ZnO-NRs with high density were grown directly over unsupported GNPs suspended in aqueous solution [18]. The morphology of the produced nanomaterials was investigated through high-resolution field emission scanning electron microscopy (FE-SEM) (Fig. 1). Figure 1a shows the pristine GNPs having thickness in the range of 2–10 nm and average lateral dimensions in the range of 1–10 µm. Figure 1b shows the hybrid ZNG nanomaterial composed by GNPs and in situ grown rod shaped ZnO-NRs. ZnO-NRs having average diameter of ~36 nm and length in the range of 300–400 nm are directly grown over the planar shaped pristine GNPs with high density. Superior crystallinity and chemical purity of these nanomaterials were systematically investigated and results are found to be identical to our earlier works [18]. It resulted out that the ZNGs are composed by hexagonal wurtzite crystalline ZnO and crystalline graphitic carbon compounds. No other impurities were detected thus ensuring the purity of the produced ZNG nanostructures. Aqueous colloidal suspensions of ZNGs were prepared through the dispersion of ZNG powder in ultrapure and sterilized deionized water using probe ultrasonication. The homogenous suspensions were then readily transferred to 50 mL sterilized centrifuge tubes.

### Bacterial strains and media

*Pseudomonas aeruginosa* ATCC 15692 and *Staphylococcus aureus* ATCC 25923 were the bacterial strains used in this study. They were grown in LB (Luria–Bertani) broth at 37 °C.

### Cells viability test

Viability was evaluated in both suspensions and solid substrates. For liquid assay, bacteria were incubated at 37 °C under gentle shaking in H_2_O_dd_ suspensions of ZNGs at various concentrations (ranging from 0.1 to 50 µg/mL). The bacterial concentration inoculated was 5 × 10^7^ cells/mL. Both microbial strains were exposed to increasing concentrations of ZNGs and compared to the respective untreated controls. The experiments were carried out at 2 and 24 h of treatment.

In the case of antimicrobial test on solid surfaces, ZNGs applied on plywood samples (2.5 cm × 2.5 cm) covered or not by a commercial paint were drop casted with 150 µL of a ZNG suspension (250 µg/mL) and air-dried. After a 30 min of UV-sterilization, 200 µL of *S. aureus* suspension (6 × 10^5^ cell/mL) were spotted onto the plywood surfaces. Cells were extracted at the initial time of contamination (t0) and after 4 h of incubation at 25 °C by washing plywood substrates in a sterile bag with 10 mL of sterile H_2_O_dd_.

The ability of bacterial survival was assessed by the colony count method (Colony Forming Unit, CFU) for both types of tests, by spreading the diluted samples onto LB agar plates.

### Evaluation of biofilm formation

The biofilm growth in 96-well microtiter plate was estimated by using the Crystal Violet (CV) assay. In the case of *S. aureus*, each well was inoculated with 200 µL of a suspension containing *S. aureus* cells (final concentration 1 × 10^7^ cell/mL), the Tryptic Soy Broth medium (TSB, Becton–Dickinson and Company, Franklin Lakes, NJ, USA) with 2% glucose (to stimulate biofilm formation) and ZNGs, present or not at various concentrations (in triplicate). For *P. aeruginosa,* 100 µL of a suspension of LB broth and ZNGs inoculated with bacterial aliquot (0.5 OD_600_) were placed in every well. After incubation of the plates under stirring (25 rpm) at 37° C for 24 h, the culture medium was removed and the wells were washed twice with H_2_O_dd_ with the purpose to remove cells not adhered. Plates were then kept at 65 °C for 20 min. Finally, every well was stained with 0.3% Crystal Violet (Sigma-Aldrich) and incubated at RT for 15 min. After several washes with H_2_O_dd_, plates were left to dry and wells were then treated with 200 µL of 96% EtOH for CV elution. Absorbance at 600 nm was then measured by using a multiplate reader (Promega, GloMax multi+ detection system).

### Pyocyanin assay in *P. aeruginosa*

For this test, 12-well microtiter plates were used. Each well was filled with 900 µL of LB broth containing or not different concentration of ZNGs (in triplicate) and inoculated with *P. aeruginosa* cells at a final concentration of 5 × 10^7^ cell/mL from an overnight growth culture, reaching a final volume of 1.5 mL (adding sterile H_2_O_dd_). Plates were incubated at 37 °C overnight without agitation. Next, ON cultures were centrifuged and the supernatant absorbance was measured at 380 nm.

### Preparation of bacterial cells for FE-SEM imaging

Treated and untreated cells of *P. aeruginosa* were incubated at 37 °C for 1 h, while *S. aureus* ones for 30 min. Short treatment times were chosen to obtain images in which the effects of ZNGs on bacterial cells were clearly visible. The tested concentration of ZNGs was 50 µg/mL in 1 mL of sterile water. The protocol for samples preparation was performed as described in Olivi et al. [21]. Imaging was performed using a Zeiss Auriga FE-SEM, operated at an accelerating voltage of 5 kV.

### FTIR

To investigate the antimicrobial properties of ZNGs, Fourier Transform Infrared (FTIR) spectroscopy was used. The comparison of the FTIR spectra of untreated bacterial cells and of bacterial cells treated with this nanocomposite allowed to assess whether the treatment induced alterations of the bacterial cell structure and surface components. Briefly, about 5 × 10^8^ cell/mL of overnight grown cultures of *P. aeruginosa* and *S. aureus* were incubated in 1 mL of sterile H_2_O_dd_ at 37° C for 90 min under gentle agitation, with or without ZNGs (10 µg/mL). Both ZNGs concentration and time of exposure were chosen in order to have a high cellular survival and to appreciate the early structural changes in treated bacteria. Cells were withdrawn and then fixed with 1 mL of a freshly prepared 4% (v/v) formaldehyde solution. After incubation for 1 h in the dark, the samples were washed three times and the cells were initially suspended in 20 μL of H_2_O (water suspension) or of D_2_O (deuterium oxide suspension). FTIR spectra have been collected either on dried samples or on liquid samples. Dried samples were prepared by drop-casting 20 μL of a bacterial suspension onto a CaF_2_ window and then leaving the liquid suspension to air-drying. Measurements on liquids were performed by placing 50 µL of a bacterial suspension in deuterated water between two CaF_2_ windows separated by a 50 μm Teflon spacer. In both cases, FTIR spectra of untreated bacterial samples and of treated bacterial samples have been acquired and then analyzed. FTIR measurements were carried out with a Bruker Vertex 70 spectrometer equipped with a DTGS (doped triglycine sulfate) detector. During data collection the sample was at room temperature and the sample compartment was under continuum purging with dry N_2_ gas. Each spectrum is an average over 256 scans and has a spectral resolution of 2 cm^−1^. In the case of dried samples, the intensity transmitted by the CaF_2_ substrate was used as a reference to obtain the sample absorbance. In the case of liquid samples, absorbance was calculated using the intensity transmitted by the CaF_2_ cell filled with pure D_2_O as a reference.

### Method of cultivation for *C. elegans*

In this study the *C. elegans* wild type strain N_2_ was used. It was maintained at 16 °C on agar plates of the Nematode Growth Medium (NGM) covered by a layer of bacterial suspension of *Escherichia coli* OP50 as feeding source [22].

### Lifespan analysis

Nematode treatment with ZNGs was performed starting on 1-day adults or newly hatched L1 larvae, resulting from synchronized cultures that were transferred to NGM-OP50 plates with ZNGs at the indicated concentrations. Every day nematodes were placed onto freshly prepared plates and 100 µL of ZNG suspensions were distributed before worms seeding. The nematodes were monitored daily for their survival with respect to untreated nematodes, and were considered dead when there was no response to the delicate touch of a platinum wire. At least 60 nematodes per condition were used in each experiment.

### Brood size

OP50-NGM plates containing or not ZNGs were seeded with adult worms (in triplicate) and were incubated at 16 °C, allowing embryos laying. Next, each animal was transferred onto a fresh plate every day, and the number of progeny was recorded for 4 days until the worm stopped laying eggs.

### Body length analysis

Nematode larvae exposed to ZNGs starting from embryos hatching, were photographed at the indicated time points by using a Leica MZ10F stereomicroscope with a Jenoptik CCD camera. Length of worm body was determined by using the Delta Sistemi IAS software. An average of 30 nematodes were imaged on at least three independent experiments.

### Pumping rate measurements

The pharyngeal pumping rate was measured in *C. elegans* individuals exposed or not to ZNGs starting from their larval development as described in lifespan assay. About 10 worms for each experimental condition were analyzed for the number of their pharyngeal contractions during a time interval of 30 s. This analysis was repeated at the indicated time points.

### Body bending evaluation

The locomotion behavior of nematodes, treated with ZNGs starting from embryos hatching, was analyzed by body bending counting at the indicated time points. After several washes in M9 buffer to remove bacteria, nematodes were placed in 10 μL of M9 buffer allowing them to swim freely. About 10 worms for each experimental condition were monitored for the number of head thrashes within a minute.

### Statistical analysis

All experiments were performed at least in triplicate. Data are presented as mean ± SD. The statistical significance was determined by Student’s t test or one-way ANOVA analysis coupled with a Bonferroni post test (GraphPad Prism 5.0 software, GraphPad Software Inc., La Jolla, CA, USA), and defined as *p < 0.05, **p < 0.01, and ***p < 0.001.

## Results and discussion

**Fig. 2:**
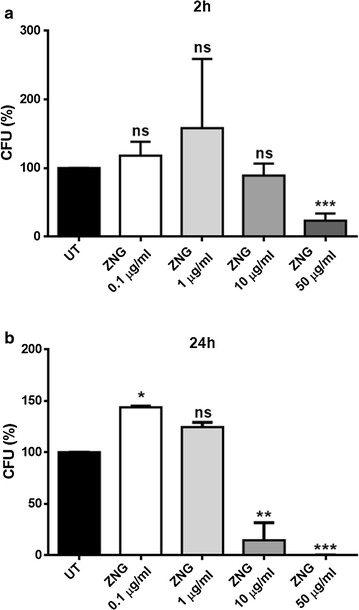
Effect of ZNGs on *Pseudomonas aeruginosa* viability. Bacteria were treated or not (UT) with different concentrations of ZNGs for **a** 2 h or **b** 24 h and bacterial survival was evaluated by CFU counting analysis. A one-way ANOVA analysis with the Bonferroni post-test was used to assess statistical significance (*ns* not significant; *p < 0.05, **p < 0.01 and ***p < 0.001 with respect to UT)

**Fig. 3:**
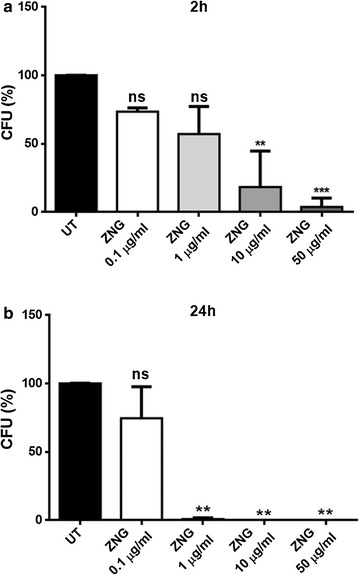
*Staphylococcus aureus* survival after exposure with ZNGs for **a** 2 h and **b** 24 h in comparison with untreated cells (UT). Statistical analysis was performed by one-way ANOVA method coupled with the Bonferroni post-test (*ns* not significant; **p < 0.01 and ***p < 0.001 with respect to UT)

In this study, the antimicrobial effects exerted by ZNGs were investigated against bacteria belonging to *Staphylococcus* and *Pseudomonas* genera, frequently found in wall paintings as biodeteriorative agents. In particular, strains of *Staphylococcus aureus* and *Pseudomonas aeruginosa* were employed to confirm the antimicrobial activity of this nanomaterial on both Gram-positive and Gram-negative bacteria, respectively. After just 2 h of treatment with ZNGs, *P. aeruginosa* revealed significant differences in bacterial survival with respect to the control when concentration of 50 μg/mL was used (Fig. 2a). Indeed, in this case only 23% of survival was observed, in contrast to a slight stimulation of cell growth showed by treatment with 1 μg/mL (Fig. 2a). Similar to short-term treatment, the 24 h-exposure to low amounts of ZNGs (0.1 and 1 μg/mL) induced a higher cell growth in *P. aeruginosa* with respect to untreated cells. By contrast, a noteworthy bacterial mortality was highlighted when ZNGs concentration was increased; exposure to 10 and 50 μg/mL led to 78 and 99.8% reduction in bacterial survival, respectively (Fig. 2b). Notably, ZNGs exerted the highest antimicrobial power against *S. aureus*, resulting already effective after 2 h of treatment at 10 μg/mL, and pointing out a 96% mortality rate at the maximum tested concentration (50 μg/mL) (Fig. 3a). In the case of long-term exposure, a strikingly elevated killer action of ZNGs was revealed even at extremely low concentrations; a mortality rate of 99.3% was indeed observed with just 1 μg/mL (Fig. 3b). In our previous studies on pristine GNPs and ZnO nanorods, the two constituents of this hybrid nanomaterial, showed a strikingly high antimicrobial power against both Gram-positive and Gram-negative bacteria including *P. aeruginosa*, *S. mutans*, *S. aureus* and *Bacillus subtilis* [20, 23, 24]. Although graphene represents an attractive material for various applications due to its unique and antimicrobial, electrical and mechanical properties (reviewed in Zhu et al. [25]), it is visibly black in colour ​as other 2D carbon-based materials. This reduces the efficiency of its use in applications where aesthetical dimension matters. The characteristic darkness of GNPs is here softened by ZnO whitening (as demonstrated in Zanni et al. [19]) and ZnO decoration also prevents GNPs aggregation, consenting, thus, its exploitation in the development of novel nanofiller-based wall paints in the field of building construction and cultural heritage. Our results are in line with the notion that a higher inhibition activity of ZnO nanoparticles has been reported against Gram-positive bacteria with respect to Gram-negative bacteria [26, 27]. After just 2 h, ZNGs treatment (10 μg/mL) resulted in 70% more antibacterial activity against the Gram-positive *S. aureus* than the Gram-negative *P. aeruginosa*. Overall, our results indicate a time- and dose-dependent bactericidal action of ZNGs against the planktonic forms of two representative Gram-positive and Gram-negative bacteria.

**Fig. 4:**
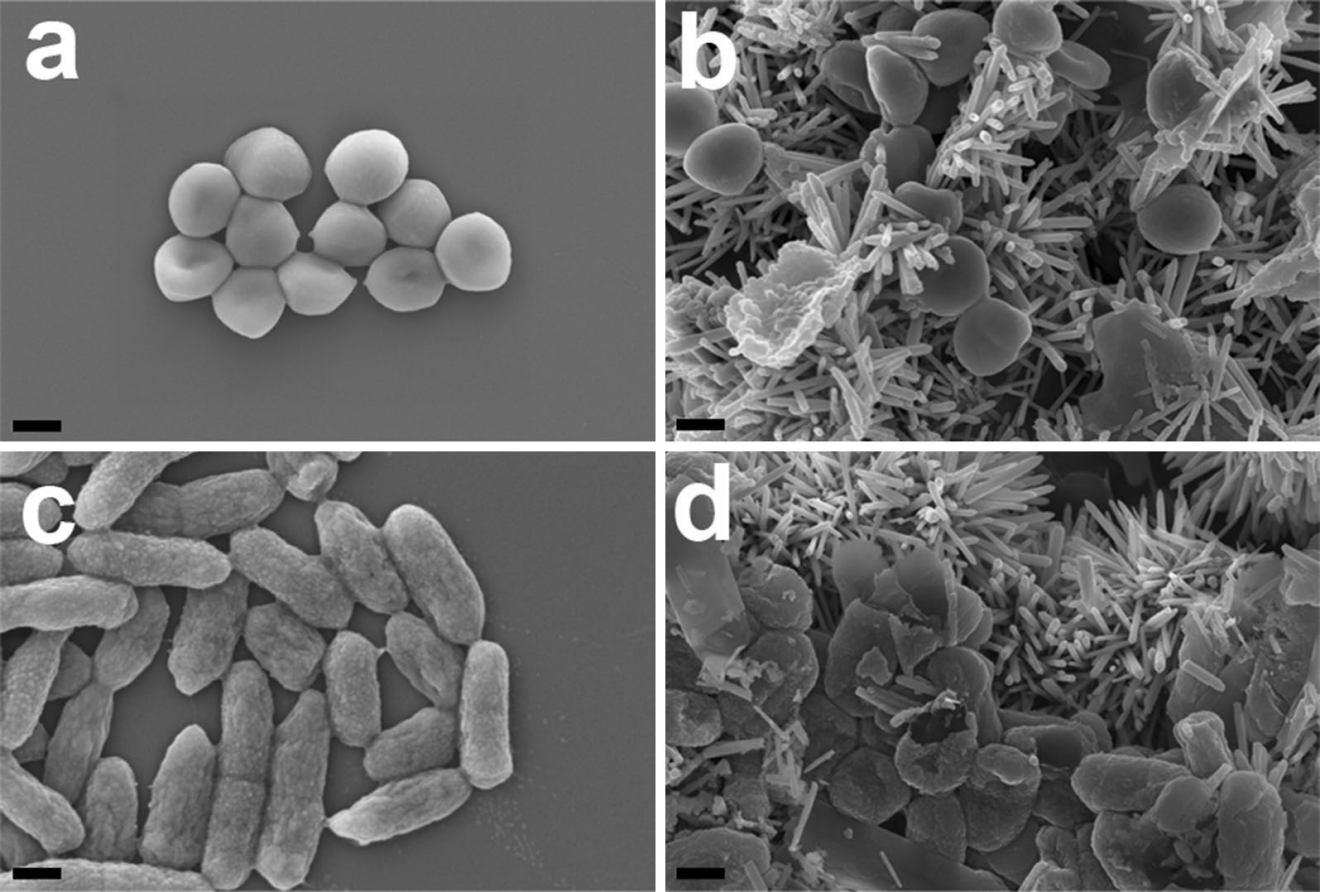
FE-SEM micrographs of bacterial cells after exposure to zinc oxide nanorods-decorated GNPs. *S. aureus* cells incubated with **a** H_2_O_dd_ or **b** ZNGs suspension (50 µg/mL). *P. aeruginosa* **c** untreated cells are shown in comparison to **d** the same bacteria exposed to ZNGs (50 µg/mL) (*scale bar* 400 nm)

Next, a FE-SEM analysis was performed in order to examine the interactions between bacterial cells and ZNGs. The untreated cells of *S. aureus* and *P. aeruginosa* resulted to be intact with their round and rod shaped morphology, respectively (Fig. 4a, c). Conversely, in treated cells the bacterial surface showed mechanical injuries caused by direct contact with ZNGs (Fig. 4b, d), which perforated the cell wall as a result of ZnO-NRs that protrude from the sheets of GNPs. Severe membrane disruption and cytoplasm leakage were observed in bacterial cells treated with ZNGs, in contrast to some cells that maintained their membrane integrity, but showing a poor living state. It can be hypothesized that nanorods adhere to cells and then act as a network of nanoneedles that pierce the bacterial wall and trap the cells, thereby inducing severe mechanical damage. On the other hand, nanosheets of GNPs offer large surface area, providing a preferred growth orientation for the ZnO-NRs over the GNP surface. Indeed, the adhesion of the nanostructures to the cell wall resulted to be improved, enhancing the penetration of the ZnO-NRs through the cell membrane. Because of their large lateral dimensions and their very sharp edges, GNPs work together with ZnO NRs to provoke mechanical injuries by acting as nanoknives, as suggested in different studies on graphene-based materials [28, 29].

**Fig. 5:**
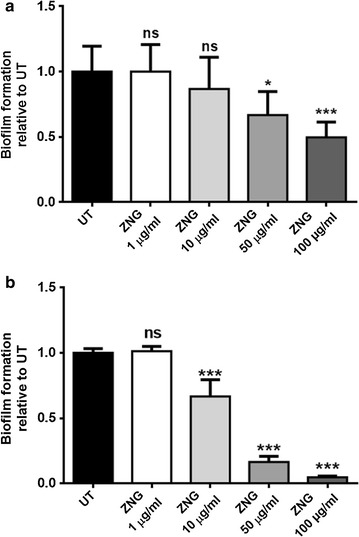
Biofilm formation was analyzed by Crystal violet binding assay in **a** *P. aeruginosa* or **b** *S. aureus* cells. The production of bacterial biomass was evaluated after exposure with the indicated concentrations of ZNGs and expressed as biofilm formation relative to untreated cells (UT). *Asterisks* indicate statistical significance (*ns* not significant; *p < 0.05 and ***p < 0.001 with respect to UT)

Furthermore, the production of pyocyanin, a virulence factor secreted by *P. aeruginosa* cells, was evaluated in bacteria exposed or not to increasing concentrations of ZNGs. The production of this bacterial blue-green pigment decreased when *P. aeruginosa* cells interacted with ZNGs; exposure to 50 and 100 μg/mL led to 50 and 70% reduction in the virulence factor secretion compared to the control, respectively (Additional file 1: Figure S1). Lee et al. demonstrated in *P. aeruginosa* that ZnO nanoparticles inhibited biofilm formation as well as the production of several virulence factors including pyocyanin [30]. Biofilm formation plays a key role as a detrimental effect in the environment in terms of biodeterioration and the spread of hospital-acquired infections. The biofilm inhibitory activity of ZNGs was thus investigated in both *P. aeruginosa* and *S. aureus* cells after 24 h of treatment. Crystal violet assay highlighted a considerable inhibitory activity of ZNGs against *P. aeruginosa* biofilm; a 13% reduction of biofilm development was observed already starting from the treatment with 10 μg/mL compared to untreated sample. Notably, the ZNG anti-biofilm activity became more evident when *P. aeruginosa* cells were exposed to 50 μg/mL, and even higher (50%) in bacteria treated with the maximum tested concentration (100 μg/mL) (Fig. 5a). Those results demonstrated the effectiveness of this nanomaterial in controlling biofilm growth of *P. aeruginosa*, one of the most wide-spread Gram-negative bacteria. Remarkably, ZNGs resulted to be stronger inhibitors of biofilm formation for the Gram-positive *S. aureus* with respect to *P. aeruginosa*. Indeed, *S. aureus* cells treated for 24 h with 10 μg/mL ZNGs, already showed a 36% decrease of biofilm formation in comparison to the untreated bacteria (Fig. 5b). Such decrease became more evident with 50 and 100 μg/mL ZNGs concentrations, showing 84 and 95% reduction in *S. aureus* biofilm development, respectively (Fig. 5b). Surface moisture is one of the main features that allow bacterial biofilm growth on different types of substrates including also architectural surfaces. Several treatments have been developed to avoid biofilm formation although biofilm removal from contaminated surfaces resulted to be not very effective [31]. Our results demonstrate that biofilm production is reduced by ZNGs treatment and that for its inhibition higher concentrations are required. This is in agreement with the notion that bacteria from biofilms are more resistant to antibacterial agents than their planktonic form [32, 33]. Recently we demonstrated that ZNGs resulted to be effective in inhibiting both the growth and the biofilm formation of *S. mutans*, the Gram-positive bacterium responsible for dental caries [19].

**Fig. 6:**
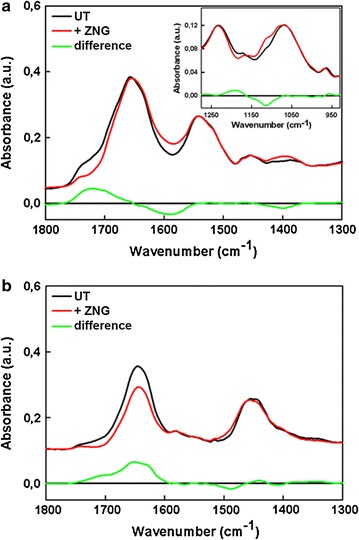
Effect of ZNGs treatment on *P. aeruginosa* cells from FTIR spectroscopy. **a** Dried samples: comparison between the untreated (UT) sample FTIR spectrum (*black line*) and the treated sample one (*red line*) in the 1800–1300 cm^−1^ spectral range. The difference between the two spectra is also reported (*green line*). Data relative to the 1300–900 cm^−1^ are shown in the inset. **b** Liquid samples (D_2_O solution): comparison between the untreated (UT) sample FTIR spectrum (*black line*) and the treated sample one (*red line*) in the 1800–1300 cm^−1^ spectral range. For the purpose of comparing the shape of different spectra, data were scaled with respect to the low wavenumbers side of the Amide II band (~1543 cm^−1^) in the case of dried samples or the Amide IIʹ band (~1450 cm^−1^) in the case of deuterated liquid samples

To assess whether the treatment induced alterations of the bacterial cell structure and surface components, the FTIR spectroscopy was employed. Figure 6 shows the FTIR spectra of *P. aeruginosa* bacteria treated or not with ZNGs for 90 min. Such choice of experimental time point was taken in order to detect the early changes in the bacterial cells due to the treatment. Several absorption bands related to dynamic properties of different functional groups of proteins, fatty acids and polysaccharides of bacterial cells are clearly visible in the FTIR spectra. In the case of dried samples (Fig. 6a) spectra in the 1800–1300 cm^−1^ is dominated by Amide I (~1656 cm^−1^) and Amide II (~1543 cm^−1^) bands, which give quantitative information on protein secondary structure [34–36]. The 1300–900 cm^−1^ spectral region (see Fig. 6a inset) contains the absorption band of phosphodiester and free phosphate functional groups (~1239 cm^−1^) and a band associated to various polysaccharides (~1090 cm^−1^) [37]. However, spectra from dried samples resulted to be characterized by low signal-to-noise ratio in the 1800–1300 cm^−^1 region. In order to put in evidence the different contributions to the line shape of the Amide I band, FTIR spectra in D_2_O solution samples were performed and are shown in Fig. 6b. For these samples, since data below 1300 cm^−1^ are dominated by D_2_O absorption bands, only the 1800–1300 cm^−1^ spectral region is reported. In this spectral range, the most prominent absorption bands are the Amide Iʹ (~1656 cm^−1^) and Amide IIʹ (which is shifted at ~1450 cm^−1^ in D_2_O) [34]. The exposure to ZnO NRs-decorated GNPs indeed clearly affects the shape and intensity of several absorption bands including the >C=O stretching of esters and of carbonic acid (1760–1700 cm^−1^) in both the dried and liquid samples indicating a modification of lipids and fatty acids content [38]. For both kind of sample preparations, it is also evident a decrease of the signal in the spectral region 1694–1675 cm^−1^ associated to “β-turns” e “antiparallel pleated β-sheets” of proteins [35, 39], suggesting changes in the secondary structure of proteins. In the case of liquid samples (Fig. 6b), we observed also a decrease in the intensity of Amide Iʹ band confirming a modification at the level of proteins secondary structure. The same spectral change is not detected when comparing the FTIR spectra of the two dried samples (Fig. 6a) probably due to the presence of residual water content in the treated sample. In the Amide II (Amide IIʹ) region essentially no change due to the treatment is detected, except for a small growth in the high wavenumbers tail of the band which could be affected by changes in the amino acid environment around the carboxylate (COO^−^) group (~1574 cm^−1^ and ~1560 cm^−1^) of aspartates and glutamates [40]. In the case of dried samples, a further evidence of such change is the increase of the signal of the carboxylate C = O symmetric stretching (~1397 cm^−1^).

In the spectral region between 1300 and 900 cm^−1^ (inset of Fig. 6a), the band at about 1234 cm^−1^, attributed to phosphodiester functional groups of DNA/RNA polysaccharide backbone structures, is essentially unaffected by ZNGs treatment. On the other hand, the band at ~1069 cm^−1^, attributed to the symmetric stretching vibration of PO_2_^−^ groups in nucleic acids and to C–O–C and C–O–P stretching vibrations of various oligo- and poly-saccharides, becomes wider because of the appearance of a component at 1114 cm^−1^. This observation suggests that an alteration in bacterial polysaccharide structures (Extracellular polymeric substances, EPS) results from the interaction of the bacterial cell surface with ZNGs, in agreement with the biofilm results. Indeed, cells forming a biofilm are surrounded by EPS, which represents the immediate environment of these cells, thus playing a relevant role in nutrient acquisition and in the protection of the bacterial cells from environment and mechanical stresses. Consistent with this, Wang et al. suggested a protective role for bacterial EPS against ZnO nanoparticles killer action, via nanostructures sequestering [41]. We can hypothesize that ZNGs act by lowering EPS production and thus by inhibiting cellular barrier mechanisms.

Fourier transform infrared spectroscopy data indicate that EPS reduction is more pronounced in *S. aureus* than in *P. aeruginosa* bacteria. This observation is in line with biofilm growth inhibition results (Fig. 5), confirming a stronger antimicrobial effect of ZnO NRs-decorated GNPs on *S. aureus*. Moreover, the FTIR spectrum of the treated *S. aureus* bacteria was, in all repeated experiments, always noisier with respect to the spectrum of the untreated ones. This result could be a further indication of the intensive interaction between the ZNGs and the external structure of the *S. aureus* bacteria. Similar overall changes (mainly alterations in the structure of proteins and polysaccharides) induced by the treatment with ZNGs are observed in the case of *S. aureus* (Additional file 1: Figure S2). However, in the 1300–900 cm^−1^ region, the band associated to saccharide structures underwent a bigger modification. Indeed, in the case of *S. aureus*, the appearance of two components, one at ~1119 cm^−1^ and the other at ~998 cm^−1^, was also observed. The FTIR results support the hypothesis that ZNGs exposure produces cell damages. In particular, the FTIR analysis suggests that the antimicrobial effect-related changes are associated with protein and phospholipid damages. This is consistent with the previously observed results demonstrating modifications in protein structures as well as membrane injuries in *S. aureus* cells treated with ZnO NRs [23]. Moreover, several studies highlighted both partial protein unfolding and changes in phospholipids as a meaning of the interaction between cell wall biomolecules and nanomaterials surface [42–44]. Cell surface proteins play important roles in cellular physiological activities, including DNA stability and replication, which in turn may lead to DNA damages.

**Fig. 7:**
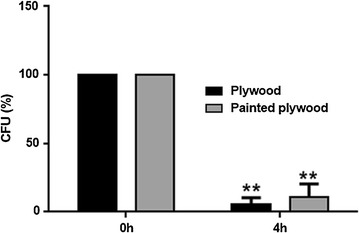
Survival of *Staphyloccocus aureus* cells on the indicated materials drop casted with ZNGs. Bacterial viability after a 4h-exposure is expressed as percentage of CFUs relative to those obtained at the initial time of contamination (t0). Data are presented as mean ± SD and asterisks indicate statistical significance (**p < 0.01)

The possible exploitation of this nanomaterial in indoor/outdoor applications prompted us to evaluate their antimicrobial properties by contaminating ZNG-decorated surfaces with *S. aureus* cells. To this aim, ZNGs were drop casted on building-related materials such as plywood sheets or samples covered by a commercial paint. ZNGs-treated surfaces induced about 95% of mortality in *S. aureus* cells already after 4 h from the contamination (Fig. 7). Overall our results demonstrate that this hybrid nanomaterial may represent a promising approach to overcome/reduce microbial growth in different application fields ranging from historical and cultural heritage to nosocomial environments and wearable medical devices.

**Fig. 8:**
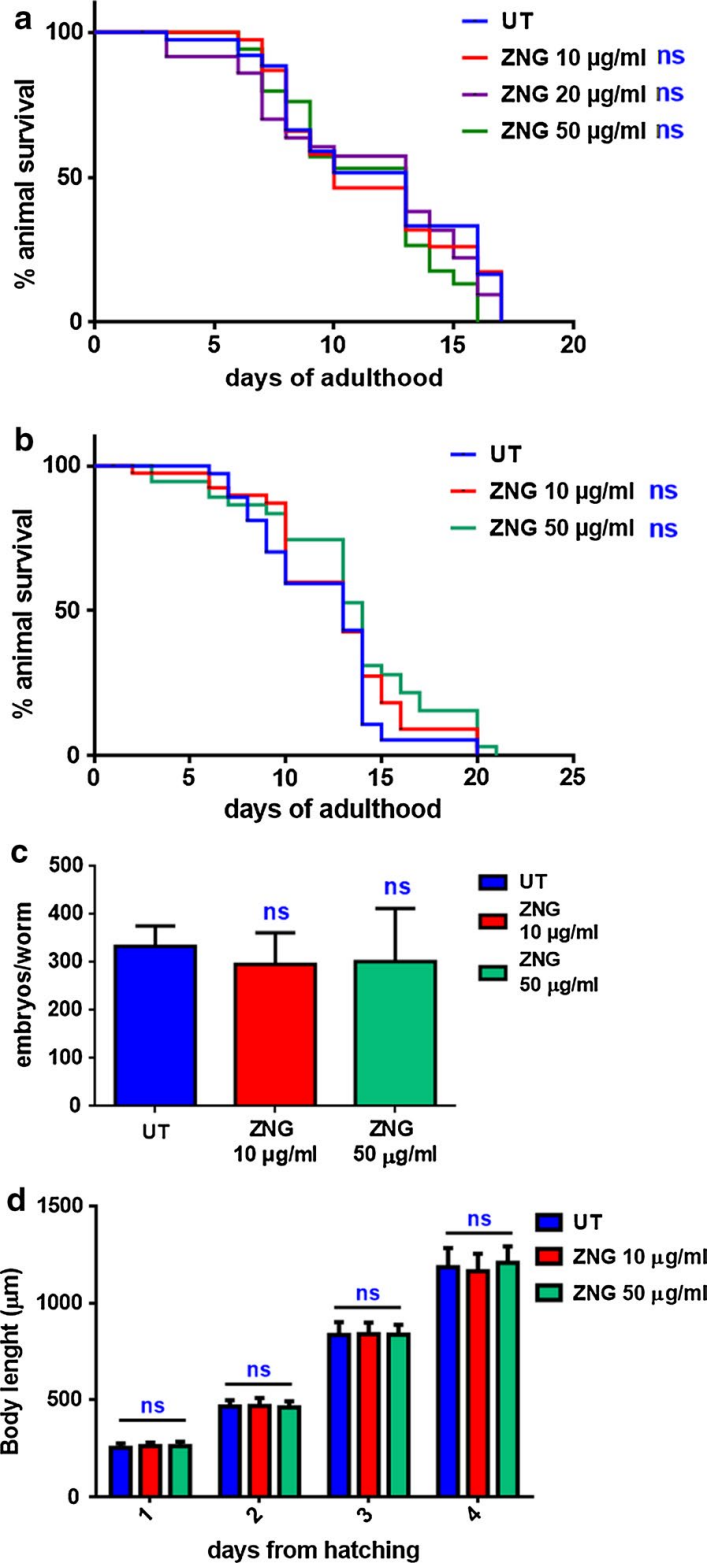
Effect of ZNGs on nematode lifespan, body size and fertility rate. Kaplan–Mèier survival plots of worms treated or not with ZNGs starting from **a** adult or **b** larval stages; *n* = 60 for single experiments. The abbreviation ‘ns’ indicates that results are not significant in comparison with control (log-rank test). **c** Average embryos production per worm of animals exposed to ZNGs with respect to untreated nematodes. Bars represent the mean of three independent experiments. **d** Effect of ZnO NR-decorated GNPs on *C. elegans* larval development. Worms were grown in the presence of *E. coli* OP50 supplemented or not with ZNGs and their length was measured from head to tail at the indicated time points. Statistical analysis of **c** and **d** was evaluated by one-way ANOVA method with the Bonferroni post-test (*ns* not significant)

The ever-growing demand for nanomaterials raise the need to understand if their environmental release could impact negatively on human healthiness. Therefore, nanotoxicity and environmental risk assessment have gained much attention in the last decade. *Caenorhabditis elegans* is a worm that has been often found in soil and leaf-litter environments, and it is emerging as a powerful model for studying neurobiology, developmental biology as well as environmental toxicology. This nematode has been extensively used to study nanotoxicity of different nanomaterials due to several features including its short lifecycle, compact genome as well as ease of maintenance (as reviewed in Gonzalez-Moragas et al. [45]). Starting from this, the biocompatibility of ZnO NRs-decorated GNPs were evaluated in the animal model *C. elegans*. Indeed, to study the effects of ZNGs on the physiology of an entire organism, several analyses were performed in worms treated with ZNGs. First, the lifespan of adult worms exposed or not to ZNGs was investigated. As shown in Fig. 8a, no significant differences were highlighted between the longevity curves of animals treated with several concentrations of ZNGs and that one relative to the control; in all experimental conditions a 50% reduction of the nematode viability was obtained around the 10th day of adulthood similarly to untreated worms, demonstrating the lack of acute toxicity in vivo.

**Fig. 9:**
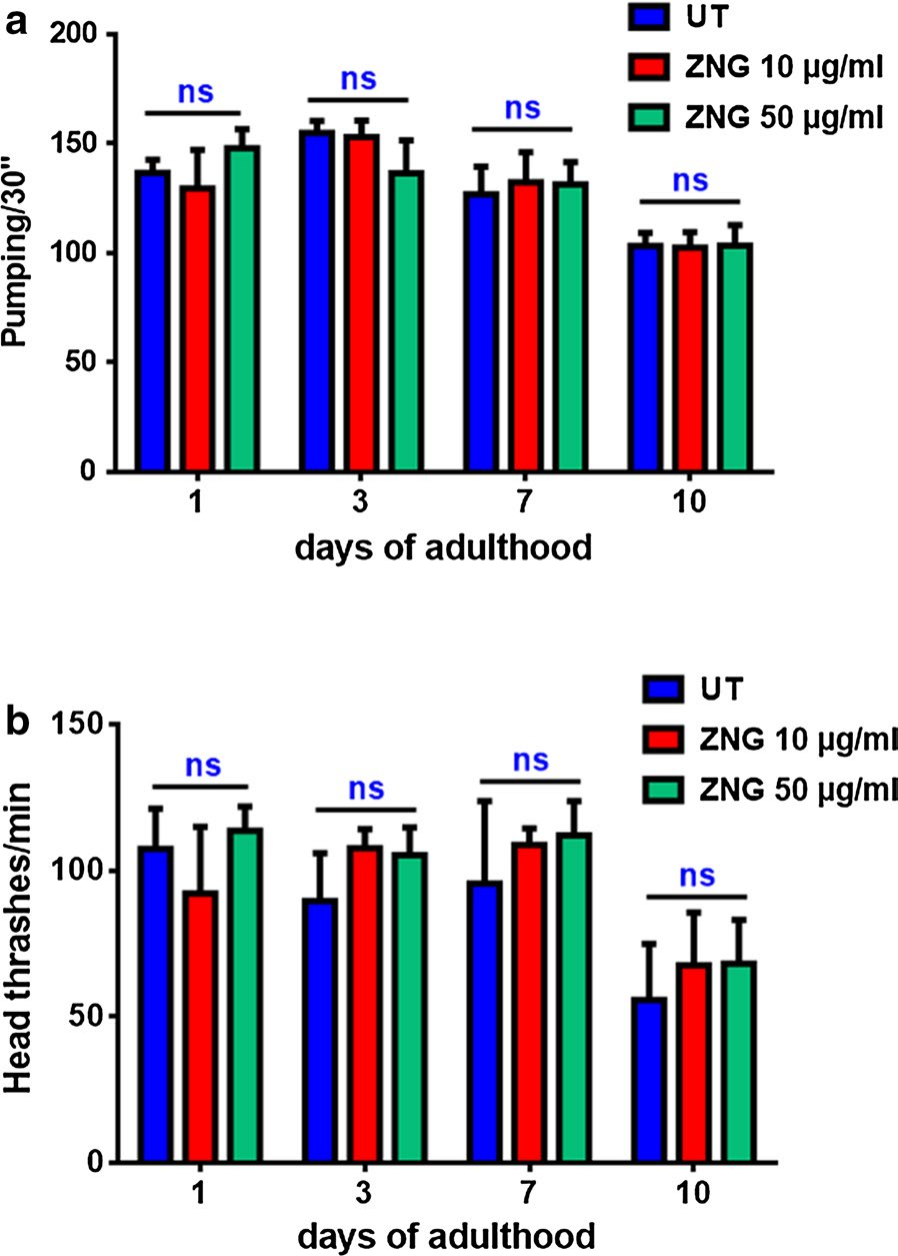
Analysis of neuromuscular functionality of *C. elegans* exposed to ZNGs. **a** Evaluation of pumping rates in nematodes treated or not with ZNGs, by measuring the number of pharynx contractions in 30 s. **b** analysis of locomotion behavior following ZNGs treatment by counting nematodes bending in the time interval of 1 min. Statistical analysis was evaluated by one-way ANOVA method with the Bonferroni post-test (*ns* not significant)

Notably, also the longevity curve of worms exposed to ZNGs starting from egg hatching resulted to be similar to that one of untreated worms (Fig. 8b), indicating that larval stage exposure to those nanoparticles did not impact negatively on *C. elegans* healthiness. Next, we evaluated the fertility rate of the ZNGs-fed nematodes as an indicator for chronic toxicity. The reproductive potential of *C. elegans* was not affected by ZnO-NRs-decorated GNPs administration to adult animals, which were able to produce an average of ~300 embryos as in the case of untreated worms (Fig. 8c); similar results were obtained when nematodes were exposed to ZNGs all along their larval development (Additional file 1: Figure S3). Then, the effect of different concentrations of ZnO-based hybrid nanomaterial was tested on *C. elegans* larval development. Indeed, after egg hatching, larvae were exposed to ZNGs and their body length was monitored every day. Even in this case, the size of treated worms did not significantly change with respect to the control at each analyzed time-point (Fig. 8d). Finally, the neuromuscular functionality of nematodes was investigated by measuring the contractions of the pharynx, a neuromuscular pump, to assess if ZNGs exposure could affect *C. elegans* swallowing ability. Even in this case, pharyngeal pumping rates were not decreased when ZNGs were administered to nematodes (Fig. 9a). In parallel, the analysis of locomotion behavior was performed to determine the impact of ZNGs exposure on *C. elegans* muscles and neurons. To this aim, the estimation of head thrashes was analyzed and resulted to be not influenced by ZNGs treatment during the first days of adulthood as well as along senescence (Fig. 9b), indicating that the exposure to ZNG did not determine negative effects on the nervous system of nematodes and hence on their motility.

We have already demonstrated that the two components of ZNGs, namely ZnO NRs and GNPs, showed non-toxic effect in *C. elegans* [23, 24]. Moreover, the lack of cytotoxicity of ZnO nanorods has been assessed in different human cell lines [38]. However, it has been reported that ZnO nanoparticles resulted to be toxic to different model systems including also *C. elegans* [39–41] and that the main components of their nanotoxicity resulted to be the reactive oxygen species production and the consequent release of zinc ion in suspensions [42]. In our previous studies we demonstrated that Zn ion dissolution was negligible in ZnO NRs as well as in ZNGs [10, 15]. We can thus speculate that the lack of ZNGs toxicity in *C. elegans* could be ascribed to the low concentration of bioavailable Zn^2+^.

Our data are consistent with the observation that the two components of ZNGs, namely ZnO NRs and GNPs, showed no harmful effects in *C. elegans* [15, 16], and that ZnO NRs resulted to be not cytotoxic in different human cell lines [46]. Although it has been reported that ZnO nanoparticles induced toxic effect in different model systems including also *C. elegans* [47–49], the main components of their nanotoxicity resulted to be the production of reactive oxygen species as well as the consequent release of zinc ion in suspensions [50]. Remarkably, in our previous studies we demonstrated that Zn ion dissolution was negligible in both ZnO NR and ZNG suspensions [19, 23], suggesting that the low concentration of bioavailable Zn^2+^ may account for lack of harmful effects in *C. elegans* exerted by of ZNGs.

## Additional file

**Additional file 1:  Figure S1.** Pyocyanin production in *Pseudomonas aeruginosa* treated or not (UT) with ZNGs for 24 h. Results are the mean of three independent experiments and error bars represent standard deviation. Statistical significance is defined as *p < 0.5 and ***p < 0.001 with respect to UT, while its absence is indicated with the abbreviation ‘ns’. **Figure S2.** Effect of graphene-ZnO nanorods treatment on *Staphylococcus aureus* from FTIR spectroscopy. **a** Dried samples: comparison between the untreated (UT) sample FTIR spectrum (black line) and the treated sample one (red line) in the 1800–1300 cm^−1^ spectral range. The difference between the two spectra is also reported (green line). Data relative to the 1300–900 cm^−1^ are shown in the inset. **b** Liquid samples (D_2_O solution): comparison between the untreated (UT) sample FTIR spectrum (black line) and the treated sample one (red line) in the 1800–1300 cm^−1^ spectral range. For the purpose of comparing the shape of different spectra, data were scaled with respect to the low wavenumbers side of the Amide II band (~1543 cm^−1^) in the case of dried samples or the Amide II’ band (~1450 cm^−1^) in the case of deuterated liquid samples. **Figure S3.** Average embryos production per worm of animals exposed to ZNGs starting from L1 larval stage with respect to untreated nematodes. Bars represent the mean of three independent experiments. Statistical analysis was evaluated by one-way ANOVA method with the Bonferroni post-test (ns not significant).
