# Supplementary material for: Evaluation of the antibacterial power and biocompatibility of zinc oxide nanorods decorated graphene nanoplatelets: new perspectives for antibiodeteriorative approaches
Source: J Nanobiotechnology. 2017 Aug 1;15:57. doi: 10.1186/s12951-017-0291-4 (PMC5539890; doi:10.1186/s12951-017-0291-4)
Supplement: Supplementary file 1 — Additional file 1: Figure S1. Pyocyanin production in Pseudomonas aeruginosa treated or not (UT) with ZNGs for 24 h. Results are the mean of three independent experiments and error bars represent standard deviation. Statistical significance is defined as *p < 0.5 and ***p < 0.001 with respect to UT, while its absence is indicated with the abbreviation ‘ns’. Figure S2. Effect of graphene-ZnO nanorods treatment on Staphylococcus aureus from FTIR spectroscopy. a Dried samples: comparison between the untreated (UT) sample FTIR spectrum (black line) and the treated sample one (red line) in the 1800–1300 cm−1 spectral range. The difference between the two spectra is also reported (green line). Data relative to the 1300–900 cm−1 are shown in the inset. b Liquid samples (D2O solution): comparison between the untreated (UT) sample FTIR spectrum (black line) and the treated sample one (red line) in the 1800–1300 cm−1 spectral range. For the purpose of comparing the shape of different spectra, data were scaled with respect to the low wavenumbers side of the Amide II band (~1543 cm−1) in the case of dried samples or the Amide II’ band (~1450 cm−1) in the case of deuterated liquid samples. Figure S3. Average embryos production per worm of animals exposed to ZNGs starting from L1 larval stage with respect to untreated nematodes. Bars represent the mean of three independent experiments. Statistical analysis was evaluated by one-way ANOVA method with the Bonferroni post-test (ns not significant). [file 12951_2017_291_MOESM1_ESM.docx]

**Additional file 1**


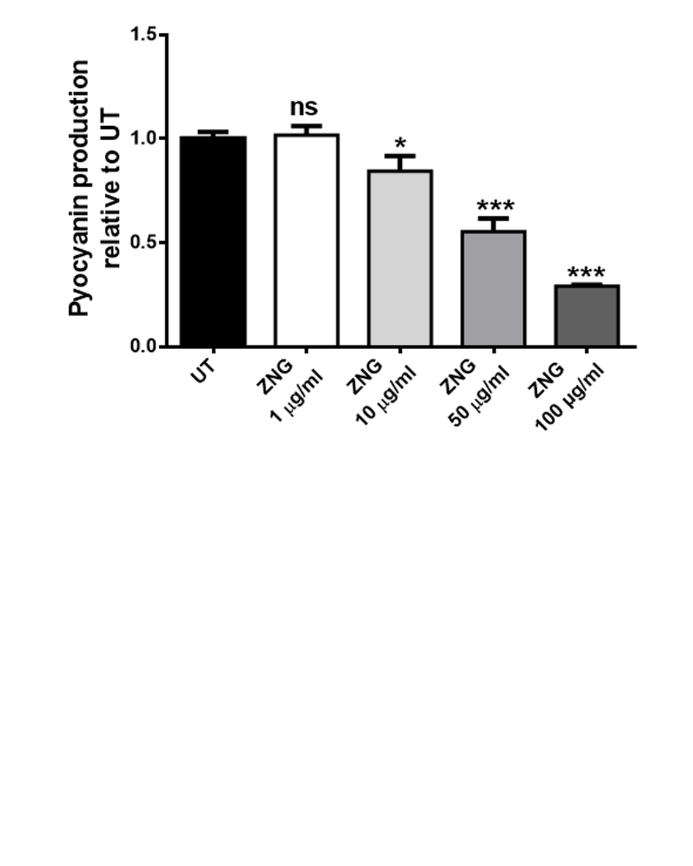


Figure S1

Pyocyanin production in *Pseudomonas aeruginosa* treated or not (UT) with ZNGs for 24 hours. Results are the mean of three independent experiments and error bars represent standard deviations. Statistical significance is defined as *p<0.5 and ***p<0.001 with respect to UT, while its absence is indicated with the abbreviation ‘ns’. The values shown in the graph represent the average of three different experiments.


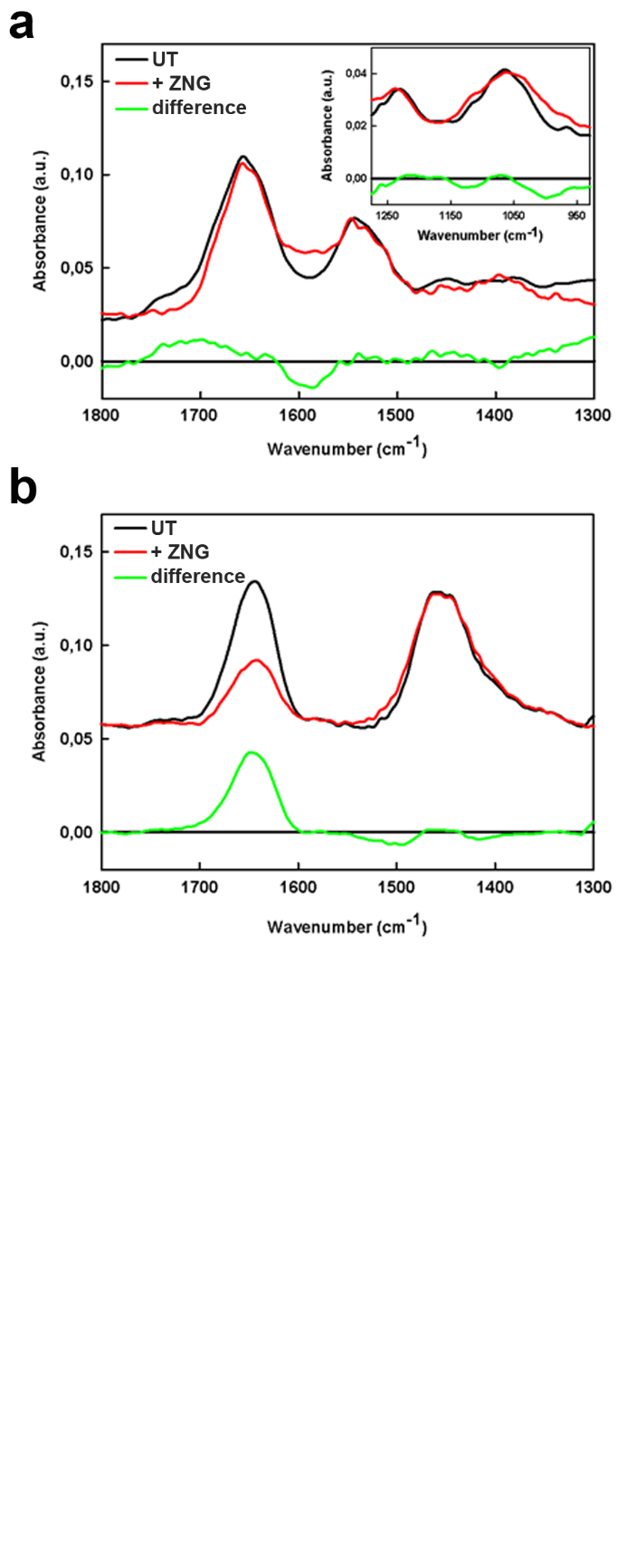


Figure S2

Effect of graphene-ZnO nanorods treatment on *Staphylococcus aureus* from FTIR spectroscopy. a) Dried samples: comparison between the untreated (UT) sample FTIR spectrum (black line) and the treated sample one (red line) in the 1800−1300 cm−1 spectral range. The difference between the two spectra is also reported (green line). Data relative to the 1300−900 cm−1 are shown in the inset. b) Liquid samples (D2O solution): comparison between the untreated (UT) sample FTIR spectrum (black line) and the treated sample one (red line) in the 1800−1300 cm−1 spectral range. For the purpose of comparing the shape of different spectra, data were scaled with respect to the low wavenumbers side of the Amide II band (~1543 cm−1) in the case of dried samples or the Amide IIʹ band (~1450 cm−1) in the case of deuterated liquid samples.


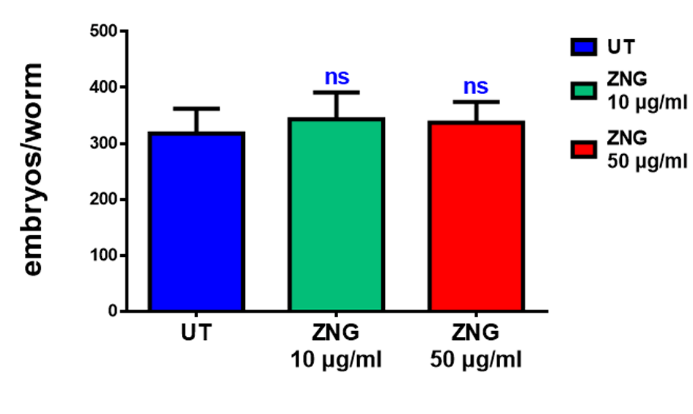


Figure S3

Average embryos production per worm of animals exposed to ZNGs starting from L1 larval stage with respect to untreated nematodes. Bars represent the mean of three independent experiments. Statistical analysis was evaluated by one-way ANOVA method with the Bonferroni post-test (ns not significant).
